# The Hydrate of Lime

**Published:** 1866-07

**Authors:** 


The Hydrate of Lime.—Liebig suggests that, in close rooms and on shipboard, deficient ventilation may be compensated by the use of hydrate of lime. Eighteen or twenty pounds of slaked lime will absorb thirty-eight or thirty-nine cubic feet of carbonic acid gas, which would be immediately replaced by an equal volume of fresh air entering through the crevices.

